# Two-year outcome of Trabeculo-Canalectomy for Chinese Glaucoma Patients

**DOI:** 10.7150/ijms.46729

**Published:** 2020-07-25

**Authors:** Zhan Xie, Zhao-Xia Mu, Mu-Long Du, Ying-Ting Zhu, Hong Sun

**Affiliations:** 1 Department of Ophthalmology, the First Affiliated Hospital of Nanjing Medical University, Nanjing, Jiangsu Province 210029, China.; 2 Department of Biostatistics, School of Public Health, Nanjing Medical University, Nanjing, Jiangsu Province 210029, China.; 3 Tissue Tech, Inc., Miami, FL, 33126, USA.

**Keywords:** trabeculo-canalectomy, glaucoma, IOP

## Abstract

To evaluate the efficacy of trabeculo-canalectomy in treating glaucoma patients, a retrospective investigation of 53 glaucoma patients (53 eyes) who underwent trabeculo-canalectomy was conducted at the First Affiliated Hospital of Nanjing Medical University, China, from April 2017 to January 2019. Intraocular pressure (IOP), visual acuity, surgical success rates, medications, and complications were monitored at post-operative 1 day, 1 week, 1, 3, 6, 12 and 24 months. Surgical success criteria were defined as 6 mm Hg≤IOP≤21 mmHg with or without additional medications. Our results showed that average IOP was statistically significant between pre-operative visit and each follow-up visit (all *P* <0.05). The total success rate of trabeculo-canalectomy at 1, 3, 6, 12 and 24 months was 92.5%, 86.8%, 94.3%, 92.5% and 90.6% respectively. After 3 months post-operatively, all patients had no obvious filtering blebs. The main early complications included postoperative hyphema (7.5%), elevated IOP (5.7%) and anterior chamber exudation (3.8%), which were all cured after conservative treatment. No blebitis, shallow anterior chamber, choroidal detachment and endophthalmitis were observed. Logistic regression analysis showed that patients with secondary glaucoma were more likely to undergo surgical failure 24 months post-operatively (*P*= 0.008). Thus, we conclude that trabeculo-canalectomy is effective and safe for the treatment of glaucoma.

## Introduction

Glaucoma is a multifactorial optic neuropathy, which causes progressive optic nerve damage and irreversible visual loss. Intraocular pressure (IOP) is considered the only modifiable risk factor in glaucoma patients. Even though a neuroprotective strategy has been developed 1, 2, lowering IOP is currently the most effective way to prevent progression of glaucomatous optic neuropathy. Surgical treatment is adopted in cases of failed medical treatment.

It was in 1968 that Cairns 3 first modified full-thickness sclerectomy into trabeculectomy. During the surgical procedure, part of the trabecular meshwork (TM) was removed, then the scleral flap tightly sutured. Consequently, the aqueous humor could drain directly into the Schlemm's canal, increasing the outflow of aqueous humor through its normal pathway. Even today, trabeculectomy remains the golden standard filtration procedure, which is applicable to almost all types of glaucoma. However, trabeculectomy is now generally regarded as an external filtration surgery depending on a functional filtration bleb, in which a small strip of corneal tissue on the limbus is removed, instead of the actual trabecular tissue. A combination of trabeculectomy and the use of anti-fibroblastic agents, laser suture lysis or releasable suture technique, offers improved efficacy for the treatment of glaucoma 3, 4. Despite continuous improvements in surgical techniques, a fraction of glaucoma patients still fail to achieve target IOP with additional topical medication and even experience trabeculectomy failure 5. Scarring of the conjunctiva and sclera is the most important factor, as the resulting fibrosis ultimately impairs the function of the filtering bleb. The study of Gedde et al. 6 showed only a 50% success rate five years after trabeculectomy over time.

With the development of modern microsurgical technology, doctors have been able to accurately position the Schlemn's canal. By removing its outer wall and the juxtacanalicular meshwork, it is possible to go back to Cairns' original idea that aqueous humor enters through the cut end of the Schlemms' canal in order to restore the natural outflow pathway of the eyeball. The growing interest in blebless surgery has led to the emergence of innovative surgical methods restoring physiological drainage 7. Our study was designed to evaluate the efficacy and safety of trabeculo-canalectomy, to build internal drainage with non-filtration blebs dependence, thus avoiding management of filtering blebs and scarring post-operatively.

## Subjects and Methods

### Ethical approval

This was a retrospective study approved by the Medical Ethics Committee of the First Affiliated Hospital of Nanjing Medical University in compliance with the *Declaration of Helsinki.* All participants were informed of the whole surgical procedures and signed informed consent.

### Patient Selection

The data of patients who underwent trabeculo-canalectomy from April 2017 to January 2019 at the First Affiliated Hospital of Nanjing Medical University were obtained from medical records. Patient characteristics are presented in **Table 1**.

### Inclusion Criteria

- Patients who failed to achieve the target intraocular pressure when given maximal IOP lowering medication or laser treatment;
- Positive diagnosis of primary open-angle glaucoma, primary angle closure glaucoma and secondary glaucoma such as traumatic and uveitic glaucoma;
- Patients failed in the routine anti-glaucoma surgery;
- Completed at least 24 months of follow-up duration postoperatively.

### Exclusion criteria

Patients with any other ocular or systemic diseases affecting vision or IOP.

### Surgical Method

**Figure 1:**
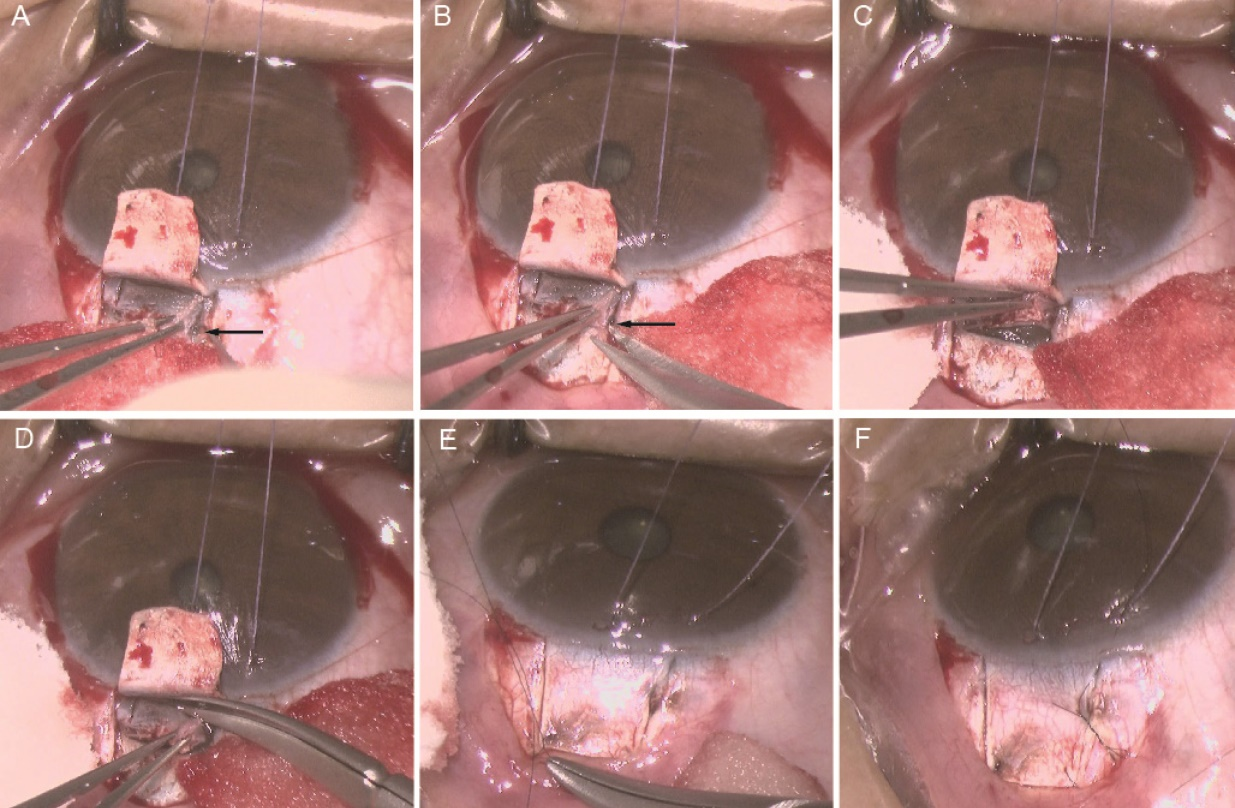
** Trabeculo-canalectomy surgical technique. A** Locating Schlemn's canal (SC); **B** Inserting the microscissor into the broken end of SC; **C** Opening the outer wall of SC; **D** Removal of the outer wall of SC and the juxtacanalicular trabecular meshwork; **E** Closing the scleral flap tightly with two sutures; **F** Closing the scleral flap with two releasable sutures. Black arrow: Schlemn's canal (SC).

All the surgeries were performed by the same surgeon. 2.5 ml of 2% lidocaine + 0.75% bupivacaine (mixed at a ratio of 1:1) was applied for peribulbar anesthesia. A suspension wire was made in the corneal limbus. A fornix-based incision through the conjunctiva and Tenon's capsule was taken, dissected through Tenon's and the episclera to bare the sclera superiorly. Gentle cautery was applied at the surgical site. A rectangular superficial scleral flap of 4 × 4 mm at the superior limbus was dissected at approximately one-half scleral thickness into the transparent cornea. Locate schlemm's canal (SC) (Fi**gure 1A**), open its outer wall (**Figure 1B,C**) and then remove the outer wall of SC as well as trabecular meshwork measuring 1 × 2 mm excluded inner wall of SC (**Figure 1D**), followed by iridectomy with Wecker scissors. The scleral flap was closed tightly with 2 sutures of 10-0 nylon (**Figure 1E**). In addition to the fixed sutures, 2 releasable sutures were placed at 2 sides of the flap (**Figure 1F**). The conjunctiva-Tenon's layer was sutured with 10-0 nylon at the ends of the incision. The corneal side port incisions were hydrated and made watertight.

### Observational Indexes

Regular follows-ups were conducted 1 day, 1 week, 1, 3, 6 , 12 and 24 months after surgery, with additional visits whenever necessary, for documentation of IOP, number of intraocular pressure-lowering drugs, best corrected visual acuity (BCVA), results of anterior and posterior segment examination, frequency of complications and postsurgical interventions and filtering bleb changes.

### Evaluation of Surgical Success or Failure

- Complete success: IOP was 6 to 21 mmHg without anti- glaucoma medications post-operatively;
- Conditional success: IOP was 6 to 21 mm Hg with local application of anti-glaucoma medications post-operatively;
- Failure: IOP was lower than 6 mmHg or higher than 21 mmHg after applying anti-glaucoma medications post-operatively. In such cases, severe eye complications were observed, for example, retinal detachment and endophthalmitis.

The height and extent of blebs are graded by Indiana Bleb Appearance Grading Scale (IBAGS) 8. Bleb height assesses the vertical dimension of the filtering bleb representing elevation of the conjunctival flap above the scleral surface and is divided into 4 scaling intervals serving as boundaries for classification: H0, flat bleb without visible elevation; H1, low bleb elevation; H2, moderate bleb elevation; and H3, high bleb as compared with the standard images. Bleb extent represents the horizontal dimension of the filtering bleb, or bleb area, and is also divided into 4 scaling intervals based on clock hours serving as boundaries for classification: E0, no visible bleb extent to less than 1 clock hour; E1, extent equal to or greater than 1 clock hour but less than 2 clock hours; E2, extent equal to or greater than 2 clock hours but less than 4 clock hours; and E3, extent equal to or greater than 4 clock hours.

### Statistics

All statistical analyses were conducted using the SPSS 21.0. Continuous variables were summarized using means, standard deviations, medians, and ranges. Categorical variables were summarized using frequencies and percentages. For continuous variables, t-test was performed for normal-distributed samples, while a corresponding non-parametric test was used for non-normally distributed samples. Repeated-measures analysis of variance (ANOVA) was used to analyze the IOP changes of patients before and after surgery. Friedman test was used to analyze the drug use changes before and after surgery. Logistic regression analysis was used for the effect evaluation of the candidate risk factors. A *p*-value of less than 0.05 was considered statistically significant.

## Results

**Figure 2:**
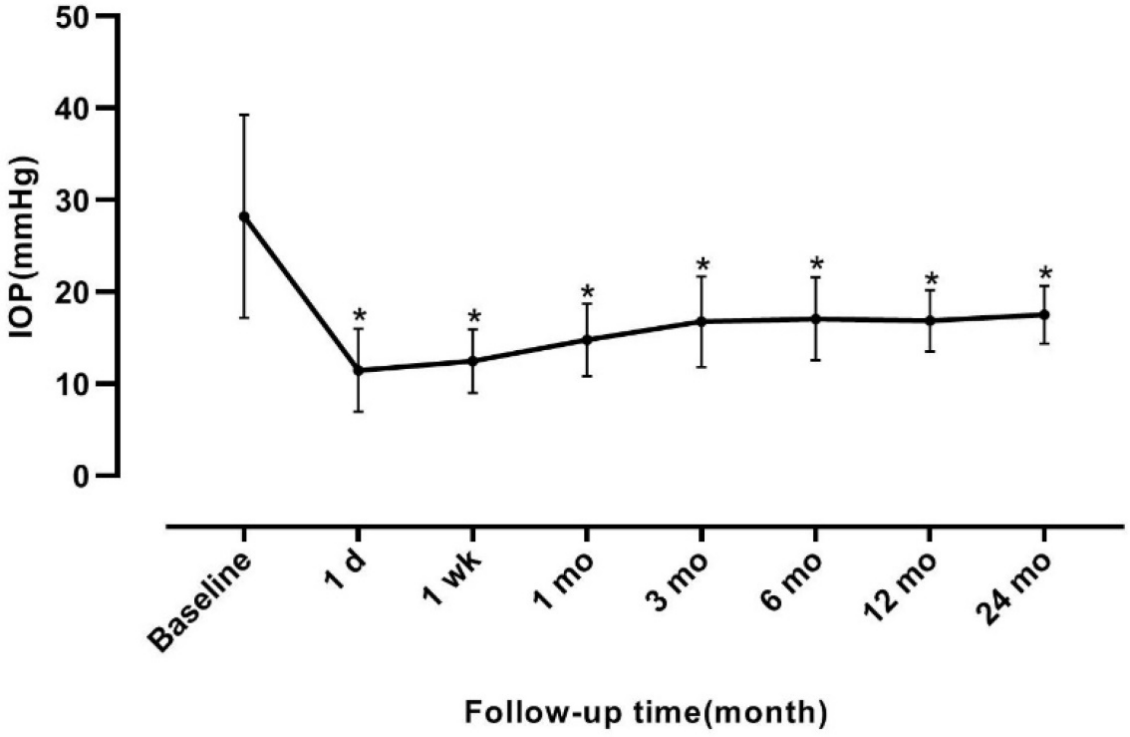
** Intraocular pressure at baseline and follow-up time.** The number of anti-glaucoma drugs before surgery was 3 (1~4). The number of anti-glaucoma drugs at 1 day, 1 week, 1, 3, 6, 12 and 24 months were 0 (0~0), 0 (0~1), 0 (0~1), 0 (0~2), 0 (0~2), 0 (0~4) and 0 (0~4), respectively. The difference between the baseline and the number of anti-glaucoma drugs at each follow-up point was statistically significant (*χ^2^*=223.191, *P*<0.001). *P<0.05 vs baseline. IOP, intraocular pressure.

A total of 53 eyes (of 53 patients) were recruited in this study. The mean age was 55.53±11.54 years (range, 20-78) and 24 of them were men. The mean pre-operative IOP was 28.23±11.05 mmHg, at 1, 7 days, 1, 3, 6, 12 and 24 months after trabeculo-canalectomy, and the mean IOP was 11.46±4.51, 12.48±3.48, 14.77±3.98, 16.77±4.92, 17.08±4.50, 16.86±3.34, and 17.52±3.14 mmHg, respectively. The difference between the mean baseline IOP and IOP at each follow-up point was statistically significant (*F*=46.871, *P*<0.001, **Figure 2**).

The total success rate in the entire study population was 90.6% at 2 years after surgery, including the complete success rate 75.1% and the conditional success rate 15.1% (**Table 2**).

**Figure 3:**
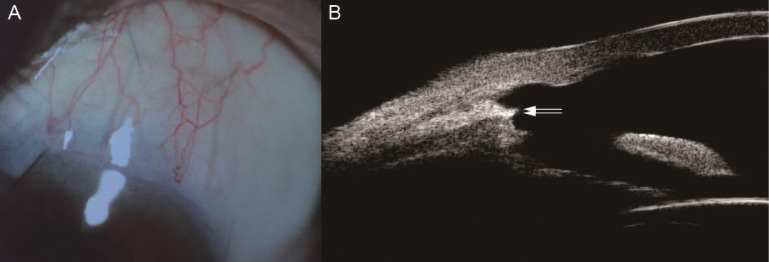
** The Filtration Bleb of One PACG Patient 3 Months after Trabeculo-Canalectomy. A** Anterior segment photograph of the filtration bleb; **B** Ultrasound biomicroscopy of the filtration bleb; PACG, Primary angle-closure glaucoma; White arrow, the inner wall of Schelemn's canal.

BCVA at 2 year post-operatively was 0.2 (0~3), which showed no significant difference compared with baseline (*Z*=-1.223, *P*=0.221). According to IBAGS, 10 (18.9%) eyes' blebs were graded as E1H1 1 week postoperatively. One month after the operation, 2 (3.8%) eyes' blebs were graded as E0H1. At 3 months post-operatively, all patients had no obvious blebs (E0H0) (**Figure 3**). Intraoperative complications included hyphema in 15 eyes (28.3%), which was controlled by oppression hemostasis with a viscoelastic agent. Four eyes (7.5%) had postoperative hyphema and 2 eyes (3.8%) had anterior chamber exudation, which was absorbed within 7 days after conservative treatment. Three eyes (5.7%) had transiently elevated IOP in post-operative 1 month, which was controlled after topical use of glaucoma medications and acupuncture separation. No blebitis, shallow anterior chamber, choroidal detachment and endophthalmitis were observed.

Single factor analysis was conducted for failure 2 years post-operatively. Factors such as age, gender, glaucoma classification, pre-operative IOP, pre-operative BCVA, pre-medications number, previous operation history, were studied. All variables that were statistically significant at *p*<0.05 in single factor analysis were included in Logistic regression model. Logistic regression analysis ascertained that glaucoma classification was the significant impact factor (**Table 3**, *p*=0.008). In consideration of model robustness, gender, age and glaucoma classification were included in the model. The results suggested that patients with secondary glaucoma were more likely to undergo surgical failure after trabeculo-canalectomy.

## Discussion

Trabeculectomy is generally considered as the gold-standard surgical procedure for glaucoma patients with medically uncontrollable IOP. However, it is plagued by a major rate of intra- and postoperative complications, such as hypotony, bleb leakage and cataract development 9. Previous studies have shown that scarring significantly affects the long-term success rate of trabeculectomy, which is the major cause of postoperative bleb failure. As a result, developing more efficient anti-scarring and non-bleb dependent surgical procedures has always been the focus to ophthalmologists. In this study, we conducted an analysis of the efficacy and safety of trabeculo-canalectomy which aims to improve aqueous outflow through conventional pathways and not to result in bleb formation.

We observed a significant reduction in IOP at 7 postoperative time points (**Figure 2**). The complete success rate of trabeculo-canalectomy at 1, 3, 6, 12 and 24 months was 92.5%, 86.8%, 94.3%, 92.5% and 90.6% respectively (**Table 2**), which showed good therapeutic effects compared with previous literature 10-12. International and domestic studies reported that IOP drop rate of trabeculectomy combined with anti-metabolizing drugs or releasable sutures in PACG patients was 44-70% 13. The success rate of trabeculectomy at 6 months postoperatively was 72%-91% 14 and 54-65% at 3 years postoperatively 15-17, which depends on follow-up time and the criteria used to define the successful outcome. In our study, no visible filtering blebs were observed in all eyes 3 months post-operatively (**Figure 3**), which fully demonstrated the efficacy of internal Schlemm's canal drainage. Since trabeculo-canalectomy is non-bleb dependent, the success rate may not be affected by external wound healing, despite the long-term outcomes need more cases and further observation.

In previous literatures, a high incidence of postoperative complications was often reported, including but not limited to shallow anterior chamber, macular edema induced by low IOP, choroidal effusion, thin-walled bleb, and endophthalmitis caused by bleb leakage 17-21. As is shown in our study, shallow anterior chamber, choroidal detachment or endophthalmitis were not observed, and there were 11 eyes (20.8%) with post-operative complications, such as hyphema, transiently elevated IOP. Overall, our surgical procedure reduced the incidence of postoperative complications, especially shallow anterior chamber. Additionally, no visible filtering blebs can avoid complications and discomfort associated with the filtering blebs. Fewer complications demonstrated the safety of our surgery.

Currently, the most common surgical techniques for glaucoma are filtering surgery and outflow channel surgery which focused on Schlemm's canal. A recent technique named canaloplasty, aimed at a circumferential catheterization with suture tensioning of Schlemm's canal, has also emerged as a safe and effective way to surgically treat POAG 22. Liang et al 23 reported that the preliminary efficacy and safety of penetrating canaloplasty in PACG patients, that the quantified success rate was 95% (19/20), and the complete success rate was 90% (18/20) at 6 months. This modified canaloplasty was performed by making a window at the corneal-scleral bed. Aqueous was redirected to the opening of Schlemm′s canal after the canaloplasty with intension sutures.

Though 360-degree catheterization of the Schlemm's canal was omitted in our study, our method also achieved satisfactory results with easier operative procedures. The most critical part of our procedure was accurately positioning Schlemn's canal. In the corneal grey-white border, the surgeon should first locate Schlemn's canal (**Figure 1A**), then deroofed its outer wall, along with adjacent trabecular meshwork (**Figure 1B-D**). After iridectomy, the scleral flap was tightly sutured with 10-0 nylon (**Figure 1E,F**), which aimed to avoid formation of external filtering blebs and restore the physiological outflow pathways of aqueous humor.

In addition, logistic regression analysis ascertained that patients with secondary glaucoma were more likely to undergo surgical failure (**Table 3**). In our study, uveitic glaucoma patients accounted for 6/7 of cases with secondary glaucoma (**Table 1**). The surgical interventions of glaucoma in uveitis have been reported challenging 24, 25 in some previous literatures, which may be due to that patients are often young, on complex medication, with intraocular scarring 26-29 and some develop superimposed steroid-induced glaucoma. Furthermore, the cutting ends of Schlemm′s canal may be more prone to the occurrence of adhesion and closure. Therefore, adequate anti-inflammatory therapy combined with the application of antimetabolic drugs was often needed.

There are several limitations to this research. Firstly, the subjects included in this study were only Chinese, thus limiting the conclusions applicable to other ethnic groups. In addition, a few patients with bilateral PACG were included in this research, which might lead to statistical bias. Further randomized controlled trials are needed to compare the efficacy and safety of our new method with traditional trabeculectomy.

## Conclusions

A target IOP around 21 mmHg is easily achievable with this procedure, with or without the use of topical medications. Trabeculo-canalectomy is an effective and safe procedure to lower IOP in patients affected by Glaucoma. Its advantages include better success rate, no bleb formation, easier follow-up and better safety profile compared with trabeculectomy mentioned in the literature, which currently is the gold standard in glaucoma surgery.

## Figures and Tables

**Table 1:** The basic data for patient enrollments

Demographics	Values
**Gender**	
Male, n (%)	24 (45.3%)
Female, n (%)	29 (54.7%)
Age (Year)	55.53±11.54
**Glaucoma Classification**	
***Primary Glaucoma***	
POAG, n (%)	7 (13.2%)
PACG, n (%)	39 (73.6%)
***Secondary Glaucoma***	
Uveitic Glaucoma, n (%)	6 (11.3%)
Traumatic Glaucoma, n (%)	1 (1.9%)
**Ocular Characteristics**	
Left Sided, n (%)	20 (37.7%)
Right sided, n (%)	33 (62.3%)
C/D Ratio	0.72±0.19
Mean Preoperative IOP-Lowing Medications, n (%)	3 (1~4)
Mean BCVA (Logmar), n (%)	0.2 (0~3)
IOP (mmHg)	28.23±11.05
**Previous History**	
Trabeculectomy, n (%)	5 (9.4%)
Cataract Surgery, n (%)	1 (1.9%)
Combined phaco-trabeculectomy, n (%)	3 (5.7%)
Non-penetrating trabeculectomy, n (%)	3 (5.7%)

N, number; POAG, primary open-angle glaucoma; PACG, primary angle-closure glaucoma; C/D, Cup/Disc; IOP, Intraocular pressure; BCVA, Best-corrected visual acurity.

**Table 2:** Post-operative results

	N	Follow-up Time (Months)	Total Success, n (%)	Complete Success, n (%)	Conditional Success, n (%)	Failure, n (%)
SUM	53	1	49 (92.5%)	49 (92.5%)	0	4 (7.5%)
	53	3	46 (86.8%)	43 (81.1%)	3 (5.7%)	7 (13.2%)
	53	6	50 (94.3%)	43 (81.1%)	7 (13.2%)	3 (5.7%)
	53	12	49 (92.5%)	40 (75.5%)	9 (17.0%)	4 (7.5%)
	53	24	48 (90.6%)	40 (75.1%)	8 (15.1%)	5 (9.4%)
Primary Glaucoma						
	46	1	43 (93.5%)	43 (93.5%)	0	3 (6.5%)
	46	3	40 (87%)	38 (82.6%)	2 (4.3%)	6 (13%)
	46	6	43 (93.5%)	36 (78.3%)	7 (15.2%)	3 (6.5%)
	46	12	44 (95.7%)	36 (78.3%)	8 (17.4%)	2 (4.3%)
	46	24	44 (95.7%)	35 (76.1%)	9 (19.6%)	2 (4.3%)
Secondary Glaucoma						
	7	1	6 (85.7%)	6 (85.7%)	0	1 (14.3%)
	7	3	6 (85.7%)	6 (85.7%)	0	1 (14.3%)
	7	6	6 (85.7%)	6 (85.7%)	0	1 (14.3%)
	7	12	5 (71.4%)	4 (57.1%)	1 (14.3%)	2 (28.6%)
	7	24	4 (57.1%)	4 (57.1%)	0	3 (42.9%)

SUM, summary; N, number.

**Table 3:** Logistic Regression Analysis of Factors influencing Surgical Success Rate at post-operative 2 Year

Factor	B	S.E.	Wald	DOF	*P*	Exp (B)
Glaucoma Classification	2.803	1.052	7.105	1	0.008	16.500
Constant	-5.894	1.635	12.992	1	0.000	0.003

B, the slope of the line; S.E, standard error; Wald, Wald test; DOF, degree of freedom; *P*,* p*-value; Exp (B), odds ratio.
